# Experiments and Observations on the Gastric Juice, and the Physiology of Digestion

**Published:** 1835-01-01

**Authors:** 


### Experiments and Observations on the Gastric Juice, and the Physiology of Digestion. By *William Beaumont*, M.D. Plattsburgh, 1833. 8vo. pp. 280.*

In our examination of the work before us, we shall endeavour to present to our readers a clear view of the facts and deductions of the author, with an occasional comment upon some of the opinions which he has advanced. We shall follow, however, a somewhat different arrangement from that adopted by Dr. Beaumont.

The subject upon which the experiments of the latter were performed, was a young man, of a good constitution, robust and healthy, who, on the 6th of June, 1822, he being then eighteen years of age, was accidentally wounded by the discharge of a musket loaded with buck-shot. The load entered his body posteriorly, and in an oblique direction, forwards and inwards, literally blowing off a portion of the integuments and muscles of the size of a man's hand, fracturing and carrying away the anterior half of the sixth rib; fracturing the fifth; lacerating the lower portion of the left lobe of the lungs and the diaphragm, and perforating the stomach. On examination, twenty-five or thirty minutes after the accident, a portion of the lung, as large as a turkey's egg, was found protruding through the exterior wound, lacerated and burnt, and immediately below this, was "another protrusion, which, on further examination, proved to be a portion of the stomach, lacerated through all its coats, and pouring out the food" that had been eaten in the morning, "through an orifice large enough to admit the forefinger."

It is unnecessary, on the present occasion, to follow out the surgical details of the accident and its treatment. For seventeen days every thing that was taken by the mouth soon passed out at the wound, and the only manner in which the patient was sustained was by nutritious injections per anum. During this period alvine evacuations could not be obtained, notwithstanding cathartic enemata were given, and various other means adopted to promote them. As soon, however, as compresses and adhesive straps could be applied over the opening into the stomach, and food was retained in the latter, by the aid of purgative injections, a very hard, black, fetid stool was procured, followed by several similar ones; after which the bowels became quite regular, and continued so.

"No sickness, nor unusual irritation of the stomach, not even the slightest nausea, was manifest during the whole time; and after the fourth week, the appetite became good, digestion regular, the alvine evacuations natural, and all the functions of the system perfect and healthy.

By the adhesion of the sides of the protruded portions of the stomach to the pleura costalis and the external wound, a free exit was afforded to the contents of that organ, and effusion into the abdominal cavity was thereby prevented."

Cicatrization and contraction of the external wound commenced in the fifth week; the stomach became more firmly attached to the pleura, but the orifice

---

* Not having been able to procure a copy of Dr. Beaumont's work in time for this Number of the Journal, we have transferred to our columns an admirable analysis of it from a recent Number of our esteemed transatlantic contemporary, the "American Journal of the Medical Sciences," under the conviction that we could not offer our readers a more instructive article or a more acceptable present. We have only omitted a few introductory criticisms not essential to the analysis or to the strictures of the reviewer.

still remained open. This resembled, in every thing but the absence of a sphincter, the natural anus, with a slight prolapsus. At every dressing it allowed the contents of the stomach to flow out, in proportion to the quantity recently taken, and when the stomach was empty, or nearly so, a partial inversion would take place, unless prevented by the application of the finger.

"Frequently, in consequence of the derangement of the dressing, the inverted part would be found of the size of a hen's egg. No difficulty, however, was experienced in reducing it by gentle pressure with the finger, or a sponge wet with cold water, neither of which produced the least pain.

In the seventh week—the circumference of the external wound was at least twelve inches, and the orifice in the stomach nearly in the centre, two inches below the left nipple, in a line drawn from this to the point of the left ilium."

The food and drink taken into the stomach were prevented from escaping through the perforation by a compress and tent of linen kept on by adhesive strips.

By the sixth of June, 1823, one year from the occurrence of the accident, the injured parts were all sound and firmly cicatrized, with the exception of the perforation leading into the stomach, which was about two and a half inches in circumference. From this time the patient continued gradually to improve in health and strength, and the newly-formed integuments became more and more firm.

"At the point where the lacerated edges of the muscular coat of the stomach and intercostal muscles met, and united with the cutis vera, the *cuticle* of the external surface, and the *mucous membrane* of the stomach *approached* each other very nearly. They did not unite, like those of the lips, nose, &c. but left an intermediate marginal space, of appreciable breadth, completely surrounding the aperture. This space is about a line wide, and the cutis and nervous papillæ are unprotected, and as sensible and irritable as a blistered surface abraded of the cuticle. This condition of the aperture still continues, and constitutes the principal and almost only cause of pain or distress experienced from the continuance of the aperture, the introduction of instruments, &c. in the experiments, or the exudation of fluids from the gastric cavity."

Compresses and bandages were constantly demanded, to prevent the escape of the food from the stomach, until the winter of 1823-4; at this period a small fold or doubling of the inner coats of the stomach appeared, forming at the superior margin of the orifice, slightly protruding, and increasing in size until it filled the aperture This valvular formation adapted itself to the opening into the stomach, so as completely to prevent the efflux of the gastric contents when the stomach is full, but was easily depressed by the finger. When the stomach is empty it plays up and down simultaneously with the respiratory muscles.

In the spring of 1824 the individual had perfectly recovered his natural health and strength. The aperture in the stomach still remained, but the surrounding wound was firmly cicatrized to its edges. From this period to the present time he has enjoyed general good health. He has been active, athletic, and vigorous; exercising, eating and drinking like other healthy and active people. For the last four months, (of the autumn of 1833,) he has been unusually plethoric and robust, though constantly subjected to a continued series of experiments on the interior of the stomach; allowing to be introduced or taken out, at the aperture, different kinds of food, drinks, various instruments, and the different contents of the stomach, almost daily, and sometimes hourly.

The perforation through the coats of the stomach is situated about three inches to the left of the cardia, near the left superior termination of the great curvature. On pressing down the valve when the stomach is full, the contents flow out copiously.

"When the stomach is nearly empty and quiescent, the interior of its cavity may be examined to the depth of five or six inches if kept distended by artificial means; and the food and drinks may be seen entering, if swallowed at this time, through the ring of the œsophagus. When entirely empty, the stomach contracts upon itself, and sometimes forces the valve through the orifice, together with an additional portion of the mucous membrane, which becomes completely inverted, forming a tumour as large as a hen's egg. After lying on the left side, and sleeping a few hours, a still larger portion protrudes, and spreads out over the external integuments, five or six inches in circumference, fairly exhibiting the natural rugæ, villous membrane, and mucous coat (?) lining the gastric cavity. This appearance is almost invariably exhibited in the morning, before rising from bed."

Dr. Beaumont commenced his first series of experiments in May, 1825; in the month of August ensuing, the young man, upon whom they were performed, returned to Canada, of which place he was a native, where he remained four years. In August, 1829, he came again to the United States, and entered into the service of Dr. B. when the latter commenced a second series of experiments, and continued them uninterruptedly until March, 1831. Soon after this period circumstances made it expedient for the subject of the experiments to return, with his family, again to Canada. In November, 1832, he once more came back and engaged himself to Dr. B. for twelve months, for the express purpose of submitting to another series of experiments, which were performed on him at Washington, and continued to March, 1833. In July of the same year, a fourth series of experiments were commenced at Plattsburgh, New York, and completed on the 1st of November, 1833.

"The usual method of extracting the gastric juice, for experiment, is by placing the subject on his right side, depressing the valve within the aperture, introducing a gum-elastic tube, of the size of a large quill, five or six inches into the stomach, and then turning him on the left side, until the orifice becomes dependent.

On introducing the tube, the fluid soon begins to flow, first by drops, then in an interrupted, and sometimes in a short continuous stream—Moving the tube about, up and down, or backwards and forwards, increases the discharge. The quantity of fluid ordinarily obtained is from four drachms to one and a half or two ounces, varying with the circumstances and condition of the stomach. Its extraction is generally attended by that peculiar sensation at the pit of the stomach, termed sinking, with some degree of faintness, which renders it necessary to stop the operation. The usual time of extracting the juice is early in the morning, before eating, when the stomach is empty and clean."

The fluid obtained in this manner, when unmixed with any thing excepting a portion of the mucus of the stomach, with which it is perhaps always combined, is clear and transparent, inodorous, a little saltish, and very perceptibly acid to the taste; having the flavour, when applied to the tongue, of thin mucilage slightly acidulated with muriatic acid. It is readily diffusible in water, wine, or spirits; slightly effervesces upon the addition of alkalies; possesses the property of coagulating albumen in an eminent degree; is powerfully antiseptic, checking the putrefaction of meat, and effectually restoring the healthy action when applied to old, fœtid sores, and foul ulcerating surfaces. When not separated by filtering, the mucus combined with the fluid, gives to it a degree of ropiness, but soon falls to the bottom in loose white flocculi. Saliva imparts to the gastric fluid an azure tinge and frothy appearance.

Equal parts of the gastric fluid and alcohol, mixed together and agitated, produced a turbid, milk-white fluid, upon the surface of which, after standing at rest, was formed a thin white coat of fine loose coagula. When the alcohol was first added to the fluid, and before the two were mixed by agitation, the latter settled to the bottom while the alcohol remained on the top, indicating that its specific gravity was less than that of the fluid.

The sensible properties of the gastric fluid are changed by a variety of circumstances; as by the admixture of saliva, water, mucus, and occasionally bile, perhaps, also, pancreatic juice. Derangement of the digestive organs, slight febrile excitement, fright, or any sudden emotion of the mind, occasions, also, material alterations in its appearance. Excess in eating causes a rancid state of the fluid, by which its solvent action is retarded. Dr. Beaumont conceives, however, that the special solvent itself—*the gastric juice*—is, probably, "invariably the same substance." The correctness of this latter opinion, the experiments before us are far, however, from establishing. It would be an interesting inquiry, which we are somewhat surprised Dr. B. has never thought of instituting, to ascertain whether the composition of the gastric juice is not varied according to the kind of aliment to which the individual is confined. According to MM. Chaussier, Virey, Pinel, and Voisin, the properties of the solvent fluid secreted by the stomach differ in different classes of animals, and in the human subject at different periods, and that this difference has a direct relation to the nature of the food. The first mentioned gentleman states, that its acidity is the greatest in herbivorous animals, the least in the carnivorous.

In regard to the composition of the gastric fluid, a portion examined by Professors Dunglison and Emmett was found to contain free *hydrochloric* and *acetic* acids, *phosphates* and *hydrochlorates*, with bases of *potassa, soda, magnesia,* and *lime*, and an animal matter soluble in cold water, but insoluble in hot. The existence of free *hydrochloric* acid in the gastric fluid was also evinced in the portion examined by Professor Silliman; in all other respects, however, the analysis of the latter gentleman is any thing but satisfactory.

The result of Professors Dunglison and Emmett's analysis corresponds very nearly with that of Tiedemann and Gmelin, who found the gastric fluid to contain, besides mucus, osmazome, and salivary matter, hydrochloric and acetic acids, alkaline sulphates and hydrochlorates, the alkali being chiefly soda; phosphate and muriate of lime and other salts in minute proportions.

Leuret and Lassaigne state the component parts of gastric juice to be water, hydrochlorate of ammonia, chloride of sodium, mucus, an animal principle soluble in water, phosphate of lime and lactic acid; they deny, however, the existence in it of free hydrochloric acid.

Now, as the lactic acid of Leuret and Lassaigne has been shewn by Berzelius to be merely a variety of the acetic, the existence of the latter in gastric juice may be considered as settled; while the researches of Prout, Children, Graves, Tiedemann and Gmelin, borne out as they are by the analysis of Dunglison, Emmet, and Silliman, establish likewise, we conceive, beyond the possibility of doubt, the presence of the hydrochloric acid in a free state.

The solvent power of the gastric juice, in relation to which so much doubt and uncertainty have heretofore existed, is proved in the most conclusive manner by Dr. Beaumont. It can never again become a subject of dispute. Almost every variety of alimentary matter, whether animal or vegetable, when submitted to the action of the fluid taken from the stomach, and kept at a temperature of about 100° Fahrenheit, was found to become, in a few hours, completely softened and reduced to a paste, resembling very nearly the contents of the stomach a short period after the same kinds of aliment had been eaten. The rapidity with which the substances were dissolved by the gastric fluid out of the body, was always in proportion to the purity of the fluid, and the tenderness of fibre and state of minute division of the substances submitted to its action. Milk and liquid albumen were found invariably to be first coagulated by the gastric fluid and then dissolved. The solution of only a certain proportion of any given aliment was effected by a certain quantity of gastric juice. Thus it was found, in many experiments, that the articles submitted to the action of the fluid taken from the stomach became softened or dissolved to a certain extent, when all further change would cease; but when more gastric juice was added, the process of solution would again commence. Cold gastric juice was found to be almost entirely inert. In one experiment, a piece of roasted beef was submitted to the action of the fluid placed in the open air at a temperature of 34°; after 24 hours it was not in the least dissolved. The temperature of the fluid being now raised to 100°, the process of solution commenced and advanced regularly.

A curious fact is shown by the experiments of Dr. B.; that food, namely, taken from the stomach a short time after it has been eaten and thoroughly mixed with the gastric juice, will become completely dissolved, provided it be kept at a temperature of 100°.

Dr. B. has found that the gastric fluid undergoes little or no change when kept in vials for a length of time. On the 1st of November, 1833, he added to one ounce of the fluid taken from the stomach eleven months before, and which was as pure as when first extracted, thirty grains of lean mutton, boiled and masticated. The whole being placed in the axilla for six hours, sixteen grains of the meat became dissolved; the solution presenting the usual appearance of chyme.

The period, as well as the quantity of gastric juice required for the solution of different alimentary substances out of the body varied, as we have already remarked, according to the density of their texture, and their state of division. Sago and tapioca, boiled, were dissolved completely in about three hours and 15 minutes; fresh wheat bread in 4 h. 30 min.; milk, boiled, in 4 h. 15 min.; unboiled, in 4 h. 45 min.; gelatine, boiled, in 4 h. 45 min.; hard-boiled eggs, in 8 h.; soft-boiled, in 6 h. 30 min.; oysters, raw and entire, in 7 h. 30 min.; stewed, in 8 h. 25 min.; beefsteak, in 8 h.; boiled beef, in 9 h. 30 min.; raw pork, in 8 h. 30 min.; fresh mutton, boiled, in 8 h. 30 min.; beef suet, boiled and entire, in 12 h.; mutton suet, boiled and divided, 10 h.; cream, 25 h. 30 min.; olive oil, 60 h.; apples, raw and entire, 18 h.; masticated, 8 h. 30 min.; turnips, boiled and entire, 13 h. 15 min.; raw, 18 h.; boiled potatoes, entire, 14 h.; mashed, 8 h. 30 min.; boiled parsnips, mashed, 6 h. 45 min.; entire, 13 h. 15 min.; raw and entire, 16 h.; raw cabbage, masticated, 12 h. 30 min.; boiled, 20 h.; mellow peach, cut small, 10 h.; mashed 6 h. An entire portion of boiled tendon required 24 h. for its solution; when masticated, 12 h. 45 min.; a portion of boiled cartilage, divided, 12 h.; masticated, 10 h; and a solid piece of bone, boiled, 80 h. In the above experiments the quantity of gastric juice employed was one ounce nearly to a drachm of the article submitted to its action.

By the above statement it will be seen that fat and oily food was among the articles which presented the greatest resistance to the solvent powers of the gastric fluid; this Dr. B. found to be invariably the case, as well in the stomach as out of it. Some of his experiments would seem to indicate that the digestibility of this species of food is facilitated by a slight admixture of bile with the gastric juice, and that, very generally, when aliment containing any quantity of fat is eaten, bile is very generally found in the cavity of the stomach.

We felt extremely desirous of comparing the observations of our author in relation to the changes produced in the healthy process of digestion, upon the different alimentary substances, with those of Tiedemann and Gmelin, by whom this subject has been examined with uncommon care and minuteness; but the want of precision in the description given of those changes by the former, and the entire absence of any thing like chemical analysis, prevent this from being done in a manner calculated to lead to satisfactory results. Taking, however, the articles albumen, gelatine, new cheese and bone, we shall give first the observations of the German experimenters, and then subjoin those of Dr. Beaumont.

Tiedemann and Gmelin found, that in the natural process of digestion, *liquid*

*albumen* forms a homogeneous fluid, in which the albumen remains entirely unchanged; this species of chyme, they remark, passes the pylorus more rapidly than any other. *Coagulated albumen* they found to be much more slowly dissolved; the fluid formed possessing the properties of coagulated albumen dissolved in acetic acid.

*Gelatine* they found to be converted into a clear brownish fluid, in which neither gelatine nor albumen could be discovered.

*New cheese,* according to these gentlemen, forms an opaque, dirty white fluid, which contains much animal matter, which is neither casein, gelatine, nor albumen.

*Bones,* in their experiments, formed a liquid, which contained not only animal matter, but also a large amount of lime.

The following observations in regard to the changes produced in the same substances, are derived from the experiments of Dr. Beaumont, performed, in the majority of cases, with the gastric juice out of the body; with the general statement that they resembled very nearly the changes which similar aliment was found to undergo when submitted to the natural actions of the stomach.

When gastric juice and *liquid albumen* were mixed together, they were so much alike in their appearance at first, that no change was perceptible; but in ten or fifteen minutes, small, white flocculi began to appear, floating about, and the mixture became of an opake whitish appearance. This appearance continued slowly and uniformly to increase for three hours, at which time the fluid had become of a milky appearance; the small flocculi had mostly disappeared, and a little light coloured sediment subsided to the bottom. No results are given of the action of the gastric fluid upon *coagulated albumen.*

Eight ounces of calf's-foot *jelly* alone were swallowed at 1 o'clock, p.m. The stomach being examined in twenty minutes, its contents were found to consist of gastric juice combined with the jelly, nearly all of which was in a fluid state; a few particles only of entire jelly were suspended in the fluid, with a few small, yellowish coagula floating near the surface. At 2 o'clock no appearance of jelly could be discovered. In another experiment, four ounces of pure *gelatine, (ichthyocolla,)* prepared with boiling water, were swallowed at 45 minutes past eight o'clock, a.m. At the end of 15 minutes the stomach appeared to be nearly as full as after an ordinary meal; it contained a clear fluid of the consistence of the white of an egg, composed apparently of the gelatine dissolved or diffused in the gastric juice. The two could not, however, be distinguished from each other. After the lapse of 45 minutes the stomach was found to be nearly empty, all that could be obtained from it being two drachms of a fluid, which appeared to be a mixture of gelatinous chyme, gastric juice, and mucous flocculi, more opaque and ropy than the gastric juice alone, and more acid than were the fluids of the stomach immediately before the gelatine was swallowed.

Thirty grains of *new cheese,* masticated, were put in three drachms of gastric juice, and kept in the axilla for eight hours and thirty minutes, when the vessel was found to contain a rich milky fluid, on which floated five grains of a matter consisting principally of oil combined with a soft caseous substance. The fluid had a strong acid, or peculiar acrid taste, and emitted a strong caseous smell, even stronger than the cheese itself, before the experiment.

*Bone,* after being dissolved in the gastric juice, formed a greyish-white opaque fluid, nearly of the colour and consistence of clear, thin gruel, with considerable fine brown sediment after standing at rest a while. It had a peculiar insipid, sweetish taste and smell, without the least fætor or rancidity.

The solvent powers of the gastric fluid being established, an important inquiry next presents itself; upon what, namely, do those powers depend? In other words, does the gastric juice act upon the food by virtue of certain specific properties which distinguish it from all other chemical agents, or are its solvent powers to be attributed solely to the acids and salts which it contains? The first of these propositions is assumed by Dr. B.

"The action of the stomach and its fluids on aliment is believed," he remarks, "to be *sui generis*, invariably the same in health on all kinds.

Chyme is a compound of gastric juice and aliment. It may be regarded as a *gastrite* of whatever it is combined with, varied according to the kind of aliment used.

Like all other chemical agents, the gastric juice decomposes or dissolves, and combines with a fixed and definite quantity of matter when its action ceases."

Without stopping to comment upon the absurd and inadmissible term *gastrite*, applied to the presumed chemical compound resulting from the union of definite proportions of gastric juice and the different alimentary substances, albumen, gelatine, fecula and the like, we shall merely remark, that the specific and invariable character and action of the fluid secreted by the stomach are mere assumptions, which are disproved by the very analysis of the fluid, which shews it to be a *mixture* of mucus, water, and various salts and acids, the nature and chemical action of which are well understood. Not a single experiment is adduced by the author which would lead us even to suspect that the gastric juice possesses any solvent or chemical property other than those which result from the substances which are known to enter into its composition, or that these do not vary, in their relative proportions at least, at different times.

If it can be shewn that other of the animal fluids, or even water, with the addition of one or other of the active ingredients contained in the gastric juice, will cause a solution of alimentary substances, similar to that produced by the latter, the idea of any specific action being exerted by it is completely overthrown. As early as 1783, it was stated by Carminati, that he digested veal with a little salt, in pure water at 100° Fah. and that the veal became partially dissolved. He employed the decanted liquor in similar experiments, until at length he procured, as he asserts, a fluid possessed of solvent properties, similar to those of the gastric juice; and in 1788, Struve and Maquart made an artificial solvent of a weak solution of ammonia, which had the same properties, according to their statement, as the gastric juice. But passing over these experiments, which may be considered inconclusive, we find that Tiedemann and Gmelin in 1825, found that water slightly impregnated with acetic or hydrochloric acid, as well as a weak solution of either the acetate or hydrochlorate of ammonia severally dissolved, more or less of nearly all the animal substances employed as food. Several experiments were likewise performed by Dr. Beaumont, which prove the solvent action upon food of diluted acetic and hydrochloric acids. In one of these experiments, equal portions of beef-steak masticated, were immersed in gastric juice, and in an equal quantity of a mixture of muriatic and acetic acids, reduced by the addition of water to the flavour of the gastric fluid as nearly as practicable. Both were kept by means of a sand bath at the temperature of 100° Fah.; at the end of nine hours the meat in the gastric juice was all dissolved—that in the acid mixture when filtered, left a residuum weighing nine grains, of a gelatinous consistence. The solution in the gastric juice was opaque, and of a lightish grey colour, and deposited on standing a brown sediment. That in the acid mixture was also opaque, but of a reddish brown colour, and deposited no sediment.

A similar experiment was repeated with pure dry gelatine. At the end of nine hours the gelatine in the gastric juice was entirely dissolved; that in the acid mixture when filtered, left a residuum of three grains of a gelatinous consistence. The solution in the gastric juice was opaque, and of a whitish colour, with a little fine brown sediment; that in the dilute acids was also opaque, but of a reddish brown colour, and of a thin, mucilaginous consistence, without any sediment. When an infusion of nut-galls was added to the first, it produced a rich cream-like fluid, and slowly precipitated a fine compact sediment; when added to the second, the whole formed immediately into a coarse brown coagulum. After standing a while, a large, loose, brownish sediment was precipitated, leaving a light coloured fluid, which became subsequently as white as milk, while the sediment became compact, and remained so.

The same experiment with gelatine being repeated, at the end of five hours and a half, the portion in the gastric juice was all dissolved to a mere mite, that in the acid mixture nearly so, six grains only of a gelatinous consistence, remaining. The fluid in the first was of a bluеish white colour; in the second, yellowish, or about the colour of dry gelatine. After remaining two hours and three quarters longer, the gelatine in the dilute acid was entirely dissolved, and the fluids of both were nearly similar. The addition of an infusion of nut-galls formed in each, loose light-coloured coagula. In the solution formed by the gastric juice, a compact sediment was thrown down, leaving an opaque milky fluid. In the solution formed by the acids, the coagula were not precipitated until after the lapse of forty-eight hours, forming then a compact mass with distinct particles of undissolved gelatine mixed with a dirty white-coloured, curd-like substance.

Another experiment was performed with a mixture of hydrochloric and acetic acids, diluted with water to the flavour of gastric juice. In this was immersed a portion of broiled steak, cut fine, and the same amount of steak was immersed in an equal portion of gastric juice. In six hours and three quarters, the meat in the latter was nearly all dissolved; in eight hours longer, that in the acid mixture was dissolved with the exception of a very small jelly-like mass. The two liquids now resembled each other very nearly. That from the gastric juice being opaque and of a lightish-grey colour, with a dark brown sediment on standing; that from the acid mixture was also opaque, of a reddish brown colour, but without sediment. The addition of an infusion of galls caused in the first a fine reddish brown precipitate, leaving an opaque liquor of a similar colour; in the second, a more copious precipitate, leaving a clearer and thinner, almost transparent liquor, of a yellowish colour.

It is well wnown that Montegre, in experiments performed with the saliva acidulated with vinegar, succeeded in dissolving various articles of food into a chymous pulp. Of the correctness of these experiments we have not the least doubt, having seen them repeated in this city with very similar results to those stated by Montegre, and having before us the additional testimony of a very late French experimenter,* who has shewn that the saliva, as well as the mucus of the intestines, obtained by opening the abdomen of an animal before eating, when slightly acidulated and kept at the temperature of the human body, will convert the food immersed in it for twenty-four or thirty-six hours into a greyish, perfectly homogeneous paste. That the intestinal mucus will produce changes in food very analogous to those resulting from the action of the gastric juice, is attested also by Tiedemann and Gmelin, as well as by Leuret and Lassaigne. The following experiment was performed by Dr. Beaumont. Two equal portions of saliva were acidulated to about the flavour of gastric juice, the one with acetic, the other with muriatic acid, and in each were immersed two pieces of parsnip and two of carrot, the one boiled and the other raw, each weighing ten grains. The temperature of the fluids was kept at 100° Fahrenheit. After 48 hours, the parsnip in the saliva with muriatic acid had lost four grains, the carrot nothing; the parsnip in the saliva with acetic acid had lost six grains, and the carrot four; they appeared to have been rather macerated and diffused than dissolved or digested. The two fluids and their contents were now mixed together, and after 24 hours the whole remaining mass of vegetable matter

* Benjamin Voisin de la Digestion Considerée en Genéral. Paris, June, 1833.

weighed twelve grains. The fluid appeared now a little more chymous, and was rather turbid.

It strikes us as not a little surprising that these experiments with artificial solvents did not suggest themselves to Dr. B. at a much earlier period than they were performed, (Feb. 1833,) and that when entered upon they were not more frequently repeated with different articles of food and with acid mixtures of various strength. Incomplete as they are, they, however, prove that as far as it regards its solvent properties, at least, the gastric fluid is not sui generis.

It will not do to say that the product of these artificial solutions is not identical with that resulting from the action of the gastric juice. This must be proved by a chemical analysis of the two. But even if they should be shown in this manner to differ materially, it is to be recollected that the gastric juice contains chemical agents independently of its acids, all of which are doubtless necessary in causing the solution of the different kinds of food, or perhaps of its different nutritive principles.

Having thus examined the observations of our author upon the nature and action of the gastric juice, we shall proceed next to the consideration of the various phenomena connected with the process of digestion. The opportunities he possessed for the careful study of these render his remarks in relation to them peculiarly interesting. It will be proper, however, to notice first the views of Dr. B. in regard to the uses of the saliva.

Excepting as a means of introducing food into the animal stomach, Dr. Beaumont maintains that mastication and salivation are to be considered as "perfectly non-essential to chymification." Neither, he conceives, would be necessary, could the food in any other way be introduced into the stomach in a finely divided state. The chyme produced by the action of the gastric fluid, out of the body, on food unmixed with saliva, exhibited, he remarks, the same sensible appearances, and was affected by reagents (?) in the same way, as that which was formed by food which had been previously masticated, mixed with the saliva and swallowed. Subsequently, Dr. B. admits that *mastication* "is absolutely necessary to healthy digestion," that it is to be considered "as one of the most important preliminary steps in the process." Although these different statements amount to a direct contradiction in language, yet we presume that all that is meant is that perfect comminution of the food, in whatever way it may be effected, is essential to its digestion; though we cannot conceive how the process of mastication can be studied in its effects separately from those of insalivation, excepting with the facilities possessed by Dr. B. and of these, so far as we are able to judge, from the detail of its experiments, he does not appear to have availed himself. A series of comparative observations, shewing the difference in the digestibility of substances swallowed after mastication in the usual manner, and those introduced into the stomach through the opening in a state of minute division only, would have settled the question; especially if the composition of the chyme formed in both instances had been carefully examined. Dr. Beaumont, it is true, asserts, as we have already remarked, that chyme from food mixed with the saliva and swallowed, and that produced by the action of the gastric juice without any mixture of saliva, did not differ in appearance, and was affected similarly by reagents—the results of these experiments are not given in detail, and of course we cannot judge of their accuracy. In one experiment it was found that the saliva, when added to aliment out of the body, had the effect of facilitating the putrefaction of the latter. This agrees with the observations of the recent German and French physiologists, and with those of Montegre. If we even admit that the only effect of this secretion is to induce in the food an incipient state of putrefaction, this of itself, according to our author's own shewing, would prove that, so far as it regards animal food, it has a very considerable agency in facilitating digestion, for "the digestibility of most meats," he remarks, "is improved by incipient putrefaction, sufficient to render the muscular fibre slightly tender."

The important part performed by the saliva in digestion, is proved, we conceive, by the fact of the large glandular apparatus for its secretion, with which nearly all animals are furnished; by the great quantity which is poured into the mouth during the process of mastication—far more than would be necessary, if it had no other office, as supposed by Dr. B., than to facilitate deglutition by lubricating the alimentary bolus; and by the additional fact, that in the duodenum the chyme is invariably mixed with another portion of fluid, identical almost in its composition with the saliva. No one who has examined a portion of food after it has been well masticated, and intimately combined with the fluid furnished by the glands of the mouth, but must be convinced that a very considerable change has been produced in it. I have ascertained *positively*, remarks Dr. Jackson,* that the saliva does exert a very energetic operation on the food; separating, by its solvent properties, some of its constituent principles, and performing a species of digestion. Voisin also† declares, that when the food is retained for a long time in the mouth, and intimately mixed with the saliva, it undergoes an actual change, by which its original character is no longer distinguishable. "I have seen it," he tells us, "converted into a greyish homogeneous pulp, very much like chyme." This change in the appearance of the aliment does not merely consist, he adds, in its conversion into a soft mass, by which it is rendered more easily swallowed—it is something more; the aliment experiences a commencing decomposition. In one experiment related by this author, when food, well triturated and imbued with saliva, was introduced into the small intestine of an animal, in two or three hours its chymification was as complete as if the process had been effected in the stomach. But Dr. Beaumont is not content with setting down the saliva as unnecessary to digestion, he has undertaken to prove further, that it actually impedes the solvent action of the gastric juice. "It would seem," he remarks, "from two or three of the experiments on artificial digestion, which were instituted for the purpose of comparison, that the mixture of saliva with the gastric juice rather retarded its solvent action;" and when mixed in large amount with the gastric fluid, it renders it fœtid in a few days. Were we to admit the opinion of Dr. B. to be correct, namely, that the mixture of saliva with the solvent fluid of the stomach vitiates the latter, this would be equivalent to asserting that digestion by the natural actions of the stomach is less perfect than that performed by filtered gastric juice on finely comminuted aliment out of the body. For we are to recollect that when solid food is eaten, it does not enter the stomach until it is mixed by the process of mastication, with a large quantity of saliva, and that under ordinary circumstances a portion of the latter is always swallowed, and of course mixes with the other fluids of the digestive organs. But we are persuaded, that whoever will read with attention the experiments of Dr. B., and compare them with each other, must be convinced from them alone, that so far from the saliva being "perfectly non-essential" to digestion, it performs a very important part in facilitating the process.

We shall proceed now to give a sketch of the very interesting observations of our author in regard to various particulars connected with the physiology of the stomach, from the correctness of which, we are happy to say, we shall have but few occasions to dissent.

Dr. Beaumont has proved with great clearness, that the gastric juice does not accumulate in the stomach in the intervals of digestion, as many physiologists, and Spallanzani among the number, have supposed; but is secreted only when

---

* Principles of Medicine, p. 354.

† Opera Citat. pp. 205-302.

food is admitted into the gastric cavity, or some other stimulus is applied directly to its lining membrane. This fact was pointed out long since by Chaussier, and more recently by the experiments of Tiedemann and Gmelin, and those of Leuret and Lassaigne.

When it does not contain food, Dr. B. has usually observed the stomach to be empty and contracted, the rugæ formed by its inner coats being irregularly folded upon each other, and almost in a quiescent state. The whole of the mucous membrane of the stomach when perfectly free from disease, is of a light or pale pink colour, of a soft velvet-like appearance, and covered constantly with a very thin transparent viscid mucus.

"Immediately beneath the *mucous coat* (?) and apparently incorporated with the villous membrane, appear small, spheroidal, or oval-shaped, glandular bodies, from which the mucous fluid appears to be secreted."

If the mucus covering the inner coat of the stomach be wiped off with a sponge during the period of chymification, the mucous membrane appears roughish, and at first, of a deep pink colour, but in a few seconds the follicles and fine papillæ begin to pour out their respective fluids, which being diffused over the parts from which the mucus had been removed, restore to them their peculiar soft, velvet-like appearance and pale pink colour, and the gastric juice begins to trickle down the sides of the stomach. When the mucus is wiped off during the period the stomach is empty, a similar roughness and deepened colour are produced, though in a less degree. The follicles appear to swell more gradually, and the fluids are not secreted in sufficient quantity to trickle down, as during the period of chymification.*

When the tongue is applied to the mucous coat of the stomach in the empty, unirritated state of the organ, no acid taste is perceptible, but whenever food or any other irritant is applied to the membrane so as to excite the gastric papillæ, an acid taste is immediately perceptible.

The ordinary temperature of the interior of the stomach during health Dr. B. has ascertained to be about 100° Fahr. as well in the intervals as during the process of digestion. There would appear, however, to be some difference in the temperature of different regions of the organ, it being somewhat higher at the pyloric than at the cardiac extremity. Variations in the state of the atmosphere were found in some of Dr. B.'s experiments, to affect the temperature of the stomach; a dry state of the atmosphere increasing, and a humid one diminishing it. Active exercise also was found to elevate invariably the temperature of the stomach, under all circumstances, about one and a half degrees.

When a portion of food is received into the stomach, the action of the vessels of its mucous coat becomes increased, the latter acquires a brighter red colour, the vermicular motions of the organ are excited, and the secretion of the gastric juice commences.

The latter appears to issue "from innumerable vessels, distinct and separate from the mucous follicles. These vessels, when examined with a microscope,

---

* Dr. Beaumont speaks of wiping off the mucous coat or *membrane* of the stomach, (page 107,) and of the mucous *coat* being restored, (ibid.) these are certainly only loose modes of expression; he cannot possibly have confounded the mucous tissue of the stomach with the mucus by which it is covered; and yet we might infer this from his language, especially when he speaks constantly of a villous coat independently of the mucous coat. We have marked in numerous parts of the work expressions in the highest degree inaccurate: thus, he speaks of "*nervous* or vascular papillæ" secreting the gastric juice, (pp. 103-4,) of glands constituting a part of "the erectile tissue of the stomach," (p. 58) and of the "excretory ducts of the gastric vessels," (p. 104.)

appear in the shape of small lucid points, or very fine papillæ, situated in the interstices of the follicles." The gastric fluid, according to the observations of the author, is secreted in quantities exactly proportioned to the amount, and greater or less degree of solubility of the food admitted into the stomach, excepting when more is eaten than is necessary for the wants of the system. The fluid is either absorbed by the portion of aliment in contact with the coats of the organ, or collects in small drops, and trickles down the sides of the stomach, to the more depending parts, and there mingles with the food or whatever else the stomach contains.

"In febrile diathesis, or predisposition from whatever cause—obstructed perspiration, undue excitement by stimulating liquors, overloading the stomach with food—fear, anger, or whatever depresses or disturbs the nervous system, the villous coat become sometimes red and dry, at other times pale and moist, and loses its smooth and healthy appearance—the secretions become vitiated, greatly diminished, or entirely suppressed—the mucous coat (?) scarcely perceptible, the follicles flat and flaccid, with secretions insufficient to protect the vascular and nervous papillæ from irritation.

There are sometimes found on the internal coat of the stomach, eruptions or deep red pimples, not numerous, but distributed here and there upon the villous membrane, rising above the surface of the mucous coat. (?) These are at first sharp-pointed and red, but frequently become filled with white purulent matter. At other times, irregular, circumscribed, red patches, varying in size or extent, from half an inch to an inch and a half in circumference, are found on the internal coat. These appear to be the effect of congestion in the minute blood-vessels of the stomach. There are also seen at times, small aphthous crusts in connexion with these red patches. Abrasions of the lining membrane, like the rolling up of the mucous coat (?) into small shreds or strings, leaving the papillæ bare, for an indefinite space, is not an uncommon appearance.

These diseased appearances, when very slight, do not always affect essentially the gastric apparatus; (?) when considerable, and particularly when there are corresponding symptoms of disease, as dryness of the mouth, thirst, accelerated pulse, &c. no gastric juice can be extracted, not even on the application of alimentary stimulus. Drinks received are immediately absorbed, or otherwise disposed of; none remaining in the stomach ten minutes after being swallowed. Food taken in this condition of the stomach, remains undigested for 24 or 48 hours, or more, increasing the derangement of the whole alimentary canal, and aggravating the general symptom of disease."

Dr. B. has observed that when a portion of food is received into the stomach, the rugæ of the latter gently close upon it, and, if sufficiently fluid, gradually diffuse it through the cavity of the organ, entirely excluding more during this action. The contraction ceasing, another quantity of food will be received in the same manner. It was found that when the valvular portion of the stomach in the subject of his experiments was depressed, and solid food introduced, either in entire pieces or finely divided, the same gentle contraction or grasping motion took place, and continued for fifty or eighty seconds, and would not allow of the introduction of another quantity until that period had elapsed, when the valve could be again depressed and more food put in. When the subject was so placed that the cardia could be seen, and then allowed to swallow a mouthful of food, the same contraction of the stomach and grasping of the bolus was invariably observed to commence at the œsophageal ring. Hence, when food is swallowed too rapidly, irregular contractions of the muscular fibres of the œsophagus and stomach are produced, the vermicular motions of the rugæ are disturbed, and the regular process of digestion is interrupted.

Contrary to the opinions of many physiologists, Dr. B. has ascertained that the solution of the food commences immediately after it is received into the stomach. Water, alcohol, and other fluids not containing alimentary matter in solution, pass from the stomach very soon after they are received, either by absorption or through the pylorus. Liquid albumen and albuminous fluids are first coagulated, and then dissolved by the gastric juice. Food taken in a liquid form combined with a large quantity of water, as soup, &c. is deprived by absorption of its aqueous portion before its digestion is commenced.

According to Dr. Wilson Philip, and the fact is confirmed by the experiments of Brodie, Broughton, Breschet, Edwards, and others, the digestion of the food commences first in the portion immediately in contact with the surface of the stomach, and as the thin layer of chyme there formed is removed by the muscular action of the organ, a second layer is chymified—digestion always commencing on the surface of the food. In reference to this opinion, Dr. Beaumont remarks :—

"That chymification commences on the surface of the food I have no doubt; but I apprehend this to be the case as it respects each individual portion, and not the whole mass.

When a due and moderate supply of food has been received, it is probable that the whole quantity of gastric juice for its complete solution, is secreted, and mixed with it in a short time. If a tenacious mass of food be used, the external portion of the whole quantity is first acted on, digested, and succeeding portions presented, &c. From numerous examinations of the stomach, I feel warranted in saying, at least in the human subject, that there is a perfect admixture of gastric juice and food—that the particles of food are constantly changing their relations with each other."

We would inquire, however, of Dr. Beaumont, whether he has ascertained positively that contact of the food with the coats of the stomach is not essential to its perfect digestion? The whole mass of food contained in the stomach may be pervaded by the gastric juice and solution go on equally in every part of it, but the question is, does a single particle become converted into perfect chyme that has not come in contact with the parietes of the digestive organ, so as to enable the absorbents of the latter to act upon it? From a careful consideration of all the phenomena of digestion, we feel no hesitation in asserting as our opinion, that chymification, strictly speaking, invariably takes place in that portion of the aliment which is applied to the inner surface of the stomach, and that it can take place no where else. It will not do for Dr. B. to reply that he has produced chyme by the action of the gastric juice on aliment out of the stomach, he must first show by a chemical analysis that the fully formed chyme as it passes into the duodenum, and the food after its solution, merely, by the gastric juice, are identically the same—and this he has not even attempted to do. That the absorbents of the stomach do act upon the aliment is proved by the fact, that a chylous fluid is formed by these vessels as well as by those of the intestines. This is shewn by the experiments of Leuret and Lassaigne, and more recently by those of Voisin.

Dr. Beaumont having observed a large portion of fluid in the stomach, even after a dry and solid meal had been eaten, presumes that a synthetic formation of water from its elements takes place in that organ. We need only remark that the supposition is in the highest degree improbable; whatever amount of fluid may be poured into the stomach during digestion, we have no right to refer it to any other source than the exhalants of the mucous membrane.

The stomach is not quiescent during the process of chymification. By the alternate contraction and relaxation of its transverse muscular fibres a peristaltic motion is produced, which commences soon after the food is received, and causes the latter to revolve around the interior of the gastric cavity, from point to point and from one extremity to another.

"The ordinary course and direction of the revolutions of the food," according to our author's observations, "are first, after passing the œsophageal ring, from right to left, along the small arch; thence, through the large curvature, from left to right. The bolus as it enters the cardia turns to the left, descends into the splenic extremity, and follows the great curvature towards the pyloric end. It then returns, in the course of the smaller curvature, to perform similar revolutions."

These revolutions are completed in from one to three minutes. They are, however, slower at first than after chymification has considerably advanced.

The motions of the stomach not only produce the revolutions of the food just referred to, but, by a kind of agitation or *churning* of the contents of the organ, cause the particles of the aliment to be separated from each other and intimately mixed with the gastric fluids.

"There is nothing," remarks Dr. B. "of the distinct lines of separation between the old and new food, and a peculiar central or peripheral situation of crude as distinguished from chymified aliment, said to have been observed by Philip, Magendie, and others in their experiments on dogs and rabbits, to be seen in the human stomach; at least in that of the subject of these experiments. The whole contents of the stomach, until chymification be nearly complete, exhibit a heterogeneous mass of solids and fluids; hard and soft, coarse and fine, crude and chymified; all intimately mixed, and circulating promiscuously through the gastric cavity, like the mixed contents of a closed vessel, gently agitated or turned in the hand."

We suspect, however, that this commixture of the different contents of the stomach, noticed in the experiments of our author, must, in some measure, have been owing to the manner in which he extracted them for examination; namely, "by depressing the valve within the aperture, shaking a little, and pressing upwards." The firm compression which the stomach exerts upon its contents, would, of itself, be sufficient to force the more fluid portions to the surface, and unless some such separation does take place we cannot conceive how the digested food is carried off, by the muscular actions of the stomach, through the pylorus, while that which has not undergone the process of chymification is retained. On two occasions Dr. B. would seem to admit, that the digested and undigested portions of the aliment occupy different portions of the gastric cavity. Thus, at page 142:—

"It is possible," he remarks, "that the portion (of aliment) presented at the perforation, may be in a more advanced stage of digestion than the rest of the mass, and consequently lighter, and float on the surface of the more solid portions of the food. In ordinary cases such would be found to be the case."

And again, at page 144:—

"It may be inferred from this experiment, (the 26th) that the more perfectly chymified portions of food rise to the superior part of the stomach, as suggested in a preceding observation, and are consequently exposed at the perforation, from whence parcels are taken for experiment and examination."

According to Dr. Wilson Philip's observations, when food has been taken at different times, the new is never mixed with the old. Dr. Beaumont, however, conceives that this statement is not correct, but that in a very short time the food already in the stomach and that subsequently eaten become combined.

"One thing," he remarks, "is certain, and it is capable of demonstration in the stomach of the subject of these experiments, that old and new food, if they are in the same state of comminution, are readily and speedily mixed in the stomach."

The ordinary time required for the complete digestion of the food received into the stomach, during a healthy state of the organ, Dr. B. has ascertained to be about three hours and a half. The facility of digestion is modified, however, by many circumstances, as idiosyncrasies, habit, the nature of the food and the manner in which it is prepared. Minuteness of division of the aliment and tenderness of its fibre, would appear to be the two great essentials for its speedy and easy digestion.

"Albumen, if taken into the stomach, either very slightly or not at all coagulated, is perhaps as rapidly chymified as any article of diet we possess. If perfectly formed into hard coagula by heat or otherwise, and swallowed in large solid pieces, it experiences a very protracted digestion. Fibrine and gelatine are affected in the same way. If tender and finely divided, they are disposed of readily; if in large and solid masses, digestion is proportionably retarded."

Animal fat is very quickly and invariably rendered fluid by the heat of the stomach, and, together with every species of oily food, resists for a long time the action of the digestive organ and its fluids. Dr. B. has observed that when the use of fat or oily food has been persevered in for a long time, there very generally takes place an admixture of bile with the gastric fluids, and from numerous experiments he has been led to believe that this admixture of bile has the effect of facilitating the solution of such kinds of aliment.

"Bulk is, perhaps, nearly as necessary to the articles of diet as the nutrient principle. They should be so managed that one should be in proportion to the other. Too highly nutritive diet is probably as fatal to the prolongation of life and health, as that which contains an insufficient quantity of nutriment."

Solid aliment Dr. B. has observed to be sooner disposed of by the stomach than fluid; he conceives, also, that its nutritive principles are sooner carried into the circulation. The correctness of the latter proposition is however very doubtful; the very fact admitted by the author, that exhaustion from abstinence, namely, is more quickly removed by liquid than by solid food, would certainly seem to disprove it.

An incipient state of putrefaction, sufficient to render the muscular fibre slightly tender, was found to increase the digestibility of most kinds of flesh.

Vegetable aliment, generally speaking, he discovered to be slower and more difficult of digestion than animal. Its solution in the stomach is greatly influenced, however, by division and tenderness of fibre. Crude vegetables often pass through the pylorus in an undigested state, while other food is retained and fully digested.

The thorough mastication of the food is essential to healthy digestion.

"If aliment," remarks the author, "in large masses be introduced into the stomach, though the gastric juice may act upon its surface, chymification will proceed so slowly, that other changes will be likely to commence in its substance before it will become completely dissolved. Besides, the stomach will not retain undigested masses for a long time without suffering great disturbance."

Consequently, eating too fast impedes digestion, by introducing food into the stomach in a state unprepared for the actions of that organ and of its fluids. If food, also, be swallowed too rapidly more will in general be taken into the stomach, before the sense of hunger is allayed, than can be digested with ease.

Overloading the stomach with aliment was invariably found to interfere with the regular process of chymification; a portion remaining for a long time undigested. This very soon becomes rancid or runs into acetous fermentation, and if not rejected by vomiting, causes pain and irritation of the stomach and other distressing symptoms; or it is permitted to pass into the intestines, where its presence almost invariably gives rise to colic, flatulence, or even more dangerous affections.

The reason why too large an amount of food is injurious, is supposed by our author to be, because "the quantity of gastric juice, either contained in its proper vessels, or in a state of preparation in the circulating fluids, is believed to be in *exact proportion* to the proper quantity of aliment required for the due supply of the system." Hence, if more food than is necessary be taken, a part of it must consequently remain undigested. We have no evidence, however, that the solvent fluid secreted by the stomach is furnished only in a certain amount; it appears to us more probable, that when too large a quantity of food is eaten, it causes an undue distention of the stomach, and in this manner prevents its regular and healthy actions from going on; while, at the same time, most generally the food is swallowed faster than the gastric juice is secreted, and in a state unfitted to be acted upon by it.

Condiments, according to our author, though they may at first excite the action of a debilitated stomach, yet when used habitually, never fail to produce indirect debility of that organ, and in this manner impede digestion.

"Salt and vinegar are exceptions, and are not obnoxious to this charge when used in moderation. They both assist digestion—vinegar, by rendering muscular fibre more tender—and both, by producing *a fluid having some analogy to the gastric juice.*"

Alcoholic, and Dr. B. thinks probably all artificial drinks, impede more or less the digestive process; some more so than others; "but none can claim exemption from the general charge. Even coffee and tea, the common beverages of all classes of people, have a tendency to debilitate the digestive organs." In the correctness of these opinions we most heartily and fully concur.

Our author has found, from numerous trials, that moderate exercise, so far from interrupting digestion, conduces greatly to its healthy and rapid performance. Severe and fatiguing exercise, however, always retards digestion.

It is stated by most physiologists, that during digestion the stomach becomes a centre of fluxion; but against the use of such an expression Dr. Beaumont strongly objects; it being one, as he declares, to which no definite meaning can be attached. We confess that we were somewhat surprised at this assertion; we have repeatedly employed the same expression ourselves, and really did believe that we were conveying to all our readers who were any way conversant with medical language a definite idea; namely, that more blood is determined to the stomach during the period of digestion than when the functions of that organ are not in exercise.* That the stomach really does become a centre of fluxion when digestion is going on, is proved by the observations which Dr. B. has himself recorded. He tells us that, during digestion, the action of the vessels of the mucous membrane is increased, that the colour of the latter is of a brighter red, and that a very copious secretion takes place from its follicles and papillæ—that all this is occasioned by an irritation of the membrane resulting from the presence of the food; and further, that gentle exercise increases the circulation in the vessels of the stomach and the temperature of the latter, and in this manner facilitates digestion.

"As the food becomes more and more changed from its crude to its chymified state, the acidity of the gastric fluids is considerably increased; more so in ve-

* We entirely agree with the transatlantic reviewer in the above remarks. We wonder, indeed, how any accurate observer could doubt the fact that, during digestion, the vital powers, as well as the vital fluids, are concentrated on the digestive apparatus. Let any one carefully attend to his own feelings, and to the phenomena presented in his own person, and he will soon be convinced of the truth of these observations.—*Editors of the Medico-Chirurgical Review.*

getable than *in* animal diet; and the general contractile force of the muscles of the stomach is augmented in every direction; giving the contained fluids an impulse towards the pylorus.

" It is probable that from the very commencement of chymification—from the time that food is received into the stomach, until that organ becomes empty, portions of chyme are constantly passing into the duodenum, through the pyloric orifice, as the mass is presented at each successive revolution. I infer this from the fact, that the volume is constantly decreasing. This decrease of volume, however, is slow at first; but is rapidly accelerated towards the conclusion of digestion, when the whole mass becomes more or less chymified. This accelerated expulsion appears to be affected by a peculiar action of the transverse muscles, or rather of the *transverse band,* as described by Spallanzani, Haller, Cooper, Sir E. Home, and others, in their experiments on animals. This band is situated near the commencement of the more conical shaped part of the pyloric extremity, three or four inches from the smaller end. In attempting to pass a long glass thermometer tube through the aperture, into the pyloric portion of the stomach, during the latter stages of digestion, a forcible contraction is first perceived at this point, and the bulb is stopped. In a short time there is a gentle relaxation, when the bulb passes without difficulty, and appears to be drawn forcibly, for three or four inches, towards the pyloric end. It is then released, and forced back, or suffered to rise again ; at the same time giving to the tube a circular, or rather spiral motion, and frequently revolving it completely over. These motions are distinctly indicated, and strongly felt, in holding the end of the tube between the thumb and finger; and it requires a pretty forcible grasp to prevent it from slipping from the hand, and being drawn suddenly down to the pyloric extremity. When the tube is left to its own direction, at these periods of contraction, it is drawn in nearly its whole length, to the depth of ten inches; and when drawn back, requires considerable force, and gives to the fingers the sensation of a strong *suction* power, like drawing the piston from an exhausted tube. This ceases as soon as the relaxation occurs, and the tube rises again of its own accord three or four inches, when the bulb seems to be obstructed from rising further; but if pulled up an inch or two through the stricture, it moves freely in all directions in the cardiac portions, and mostly inclines to the splenic extremity, though not disposed to make its exit at the aperture. Above the contracting band, and towards the splenic portion of the stomach, the suction or grasping motion is not perceptible, but when the bulb is pushed down to this point, it is distinctly felt to be grasped, and confined in its movements. These peculiar motions and contractions continue until the stomach is perfectly empty, and not a particle of food or chyme remains, when all becomes quiescent again.

If the bulb of the thermometer be suffered to be drawn down to the pyloric extremity, and detained there for a short time, or if the experiment be too frequently repeated, it causes severe distress, and a sensation like cramp or spasm, which ceases on withdrawing the tube, but leaves a sense of soreness and tenderness at the pit of the stomach.

These peculiar contractions and relaxations succeed each other at irregular intervals, of from two to four or five minutes. Simultaneously with the contractions, there is a general shortening of the fibres of the stomach. This organ contracts upon itself in every direction, and its contents are compressed with great force. During the intervals of relaxation, the rugæ perform their vermicular motions, and the undulatory motions of the fluids continue."

From the foregoing facts, Dr. B. draws the following conclusions: namely, that—

" The longitudinal muscles of the whole stomach, with the assistance of the transverse ones of the splenic and central portions, carry the contents into the pyloric extremity. The circular or transverse muscles contract progressively from left to right. When the impulse arrives at the *transverse band*, this is excited to a more forcible contraction, and closing upon the alimentary matter and fluids contained in the pyloric end, prevents their regurgitation. The muscles of the pyloric end now contracting upon the contents deposited there, separate and expel some portion of the chyme. After the contractile impulse is carried to the pyloric extremity, the circular band and all the transverse muscles become relaxed, and a contraction commences in a reversed direction from right to left, and carries the remaining contents again to the splenic extremity, to undergo similar revolutions."

"After the expulsion of the last particles of chyme, the stomach becomes quiescent, and no more (gastric) juice is secreted, until a fresh supply of food is presented for its action, or some other mechanical irritation is applied to the internal coat (of the organ.)"

We have inserted the preceding quotations, notwithstanding their length, in consequence of the highly interesting view which they present of the muscular actions of the stomach during digestion. The opportunity which the author enjoyed for studying them with care, precludes any doubt as to the correctness of his observations.

We come next to the consideration of a very important question; what, namely, are the changes produced in the food by the process of chymification? That solid food is dissolved in the stomach, we have now most abundant proof, and that most kinds of aliment undergo other and still more important changes, we have very strong reasons for presuming. But whether these changes consist merely in the breaking up of the union which existed between the proximate principles of the food, in the separation of such as are adapted for the formation of chyle from the recrementitious particles, or in an actual alteration in its chemical composition, are questions which still remain undecided. Not the least information in relation to them can be gleaned from the experiments and observations under review.

Chyme, or the product of stomachic digestion, is generally described to be a homogeneous, grayish paste, of a slightly acid taste; its acidity was found by Tiedemann and Gmelin to be greatest when the food is the most difficult of digestion. According to the observations of Dr. B. in its homogeneous appearance the chyme is invariable, but not in its colour, this being affected in a slight degree by the kind of food from which it is produced.

"It is always," he remarks, "of a lightish or grayish colour, varying in its shades and appearance from that of cream to a grayish or dark-coloured gruel. It is also more consistent at one time than at another; modified in this respect by the kind of diet used. This circumstance, however, does not affect its homogeneous character. A rich and consistent quantity is all alike, and of the same quality. A poorer and thinner portion is equally uniform in its appearance. Chyme from butter, fat meats, oil, &c. resembles rich cream. That from farinaceous and vegetable diet has more the appearance of gruel.—It is invariably distinctly acid, and possesses properties different from the elements of which it is composed."

A series of microscopic examinations of the chyme are furnished by the author; they lead, however, to no satisfactory conclusions in regard to its real character and composition.

It will, no doubt, be anxiously inquired, whether, by the experiments and observations of Dr. Beaumont, all the agents concerned in the process of digestion have been determined? To this inquiry, the reply must be in the negative. Excepting so far as relates to the solvent powers merely of the gastric juice, they leave every thing in relation to the efficient cause of digestion in the same doubt and obscurity in which it was previously involved. Dr. B. it is true, infers from the result of his experiments, that the gastric fluid is the sole agent, by which the food is converted into chyme; but until he shall be able to prove that fully-formed chyme, in the state in which it passes into the duodenum, and the fluid mass which results from the action of the gastric juice alone upon the food, are in all respects identically the same, and that the absorbents of the stomach do not act upon the dissolved aliment presented to their orifices, we must be permitted to consider his opinion in regard to the uses of the gastric juice as a mere hypothesis, the facts in support of which are still to be made out. Even the proposition with which the work before us closes, namely, "that *no other* fluid produces the same effect on food that gastric juice does, and that it is the *only solvent* of *aliment*," he is very far from having established. Tiedemann and Gmelin, as well as Leuret and Lassaigne, maintain, as the result of their experiments, that the mucus of the intestines possesses equally with the gastric juice the power of dissolving the food and converting it into a substance similar to chyme, and the fact is supported by the later observations of Voisin. The latter gentleman relates a number of experiments which prove that the gastric juice is not essential to the perfect digestion of alimentary substances. Of these experiments we present the following summary:—1st. Food triturated and mixed with saliva, when introduced into the small intestines of an animal, was in two or three hours as completely chymified as though the process had been performed in the stomach. 2d. Food of a moderate consistence, without any preparation, introduced into the upper portion of the small intestine of an animal, the communication between the intestine and stomach being cut off by the passage of a ligature, became perfectly chymified. Chyle as well as fæces were also formed. A dog was nourished in this manner for a month, and then killed. 3d. Food introduced into the cœcum, the ileo-cœcal valve being closed by a ligature, was, at the end of four hours, found to be sensibly changed, and presented some of the characters of chyme.

The fact is, the absorbents of the stomach and alimentary canal generally, perform a much more important part in the process of digestion than is commonly supposed. Doubtless the saliva, the gastric fluids and even the bile and pancreatic juice, all, under ordinary circumstances, facilitate in a very great degree the conversion of the food into chyme and the formation of chyle; but to no one nor to all of them are we inclined to ascribe any further agency in the process of digestion.

A number of experiments were performed by Dr. B. to ascertain, if practicable, the effects produced by the bile and pancreatic juice, when added to chyme. These experiments are acknowledged by the author to be very imperfect, and to lead to no positive conclusions. In the general summary, nevertheless, of the inferences which he conceives to be deducible from his experiments and observations is the following, namely, "that chyme is formed in the duodenum and small intestines, by the action of bile and pancreatic juice on the chyme." It is hardly necessary for us to enter into a refutation of this assertion. No physiologist, so far as we are aware, states that he has ever *seen* chyle in any part of the cavity of the intestines, while many, after performing numerous experiments to determine the fact, have declared that chyle never exists out of the lacteals, a conclusion which is now almost universally adopted. That the bile and pancreatic juice, particularly the former, are not by any means essential to the formation of chyle, is conclusively established by the facts adduced by the German and French experimenters so frequently alluded to in this review, and which, likewise, very clearly point out the manner in which Brodie and Mayo were led into the erroneous conclusion that when the choledochus duct is tied in animals no trace of chyle can be detected in the lacteals. The recent experiments of Voisin prove, also, that chyle is formed notwithstanding the obli-

F 2

teration of the common duct of the liver and gall-bladder. With these remarks we take our leave of this portion of Dr. Beaumont's work.

Before concluding, we have a remark or two to make in reference to our author's explanation of the cause of hunger. Dr. B. maintains, that the quantity of gastric juice necessary for the solution of just so much food as is required for the due support of the system is prepared during the intervals of digestion, and, just before a meal, fills and distends its proper vessels, ready to be poured into the stomach the moment food is swallowed; and that the sensation of hunger is produced by this distention or repletion of the secernent vessels of the stomach by the gastric fluid.

We might reply to this hypothesis by asking the author for the evidence by which the correctness of his premises is established. Is it established satisfactorily, that the gastric juice is secreted previously to the stimulus of food being applied to the coats of the stomach, and only in a certain definite amount? Or can it be proved that a distention of the "gastric vessels," as Dr. B. terms them, does really exist whenever the sensation of hunger is experienced, and that the intensity of the latter is in exact proportion to the degree in which these vessels are loaded with the solvent fluid? We shall certainly be excused if we refuse our assent to the author's explanation of the cause of hunger, until the above points are clearly made out. But in the absence of any fact which bears directly upon them, we conceive that from the author's own experiments the incorrectness of his views in this particular may be shown. 1st. If there is in fact an exact relation between the quantity of the gastric juice in its proper vessels, and the quantity of aliment demanded by the wants of the system, how is it possible that the subject of our author's experiments could take into his stomach a full meal, a very short time after Dr. B. had drawn off one or two ounces of the juice, and yet digestion be regularly and promptly performed without being in the least affected by the loss of so considerable a portion of the proper solvent fluid. 2dly. If hunger depend upon the distention of certain vessels of the stomach by the gastric juice, how comes it that an hour or two before the least sensation of hunger was experienced, the author was able to draw off a large amount of gastric juice from the stomach, without the appetite of the patient being prevented from occurring at his regular meal-time, while in other instances, immediately preceding a meal a very small quantity of the juice was with difficulty procured, and yet the usual amount of food being taken immediately afterwards, its digestion was effected without the slightest unusual delay or difficulty. 3dly. How does the author's theory of the cause of hunger comport with the following fact. In experiment 64, page 207, three drachms of gastric juice were extracted from the stomach, and in fifteen minutes afterwards the young man ate four ounces of pure gelatine prepared with boiling water, which was almost entirely digested at the end of an hour, when a breakfast of pork and bread was taken with the usual degree of appetite. Thus, notwithstanding the unloading of the distended vessels by the extraction of three drachms of gastric juice, and by that which was poured into the stomach to dissolve four ounces of gelatine, it appears that the ordinary natural appetite of the subject was in no degree destroyed.

In many persons appetite for food is destroyed by allowing the usual period of a meal to pass by without eating, and in most individuals it is almost instantly dissipated, and even the food already taken prevented from being digested, by sudden emotions of the mind, disgust and other sensations. These facts, it is true, may be explained in conformity with the views advanced by our author, by supposing an immediate absorption, in such instances, of the gastric juice distending the vessels, and a suspension for a time of its further secretion; but we have no evidence either that distention of the gastric vessels or absorption of the gastric juice contained in them takes place. If we were to presume that distention of the gastric vessels produces the sensation of hunger, and that when aliment is not taken into the stomach at regular periods the gastric juice is absorbed, prolonged abstinence, whatever effects it may produce upon the system, should never give rise to that craving for food, that extreme hunger, which we know is the most tormenting phenomenon by which it is attended.

There are many other points embraced in the work before us which we should like to have noticed, had our limits permitted, but we must now draw our remarks rapidly to a close.

We have presented, so far as we were able, in the space allotted to this review, a clear, and we trust satisfactory, view of the labours and opinions of the author. We have acknowledged the importance of the facts established by his experiments and observations, and given him credit for the perfect candour with which his opinions have been formed; we have taken the liberty, however, to dissent from the latter whenever we believe them to be unsupported by sufficient evidence, or in opposition to the facts already in our possession.

The experiments and observations of Dr. Beaumont cannot fail to be favourably received by the members of the profession, as affording, in very many particulars, a valuable addition to their knowledge of the physiology of certainly one of the most important organs of the animal system, and as a means of facilitating the inquiries of future experimenters into the true nature and cause of chymification.

In the event of a second edition, which will no doubt be speedily called for, a careful revision of his text will enable the author to remove those inaccuracies and obscurities of style with which the present is replete.

